# A Self-Management Intervention for African Americans With Comorbid Diabetes and Hypertension: A Pilot Randomized Controlled Trial

**DOI:** 10.5888/pcd11.130349

**Published:** 2014-05-29

**Authors:** Elizabeth B. Lynch, Rebecca Liebman, Jennifer Ventrelle, Elizabeth F. Avery, DeJuran Richardson

**Affiliations:** Author Affiliations: Rebecca Liebman, Jennifer Ventrelle, Elizabeth F. Avery, Rush University Medical Center, Chicago, Illinois; DeJuran Richardson, Rush University Medical Center, Chicago, Illinois, and Lake Forest College, Lake Forest, Illinois. Dr Lynch is also affiliated with Lake Forest College, Lake Forest, Illinois.

## Abstract

**Introduction:**

The objective of this pilot 6-month randomized controlled trial was to determine the effectiveness of an intensive, community-based, group intervention that focused on diet, physical activity, and peer support for reducing weight among urban-dwelling African Americans with comorbid type 2 diabetes and hypertension.

**Methods:**

Sixty-one participants were randomized into an intervention or control group. The 6-month intervention consisted of 18 group sessions led by a dietitian in a community setting and weekly telephone calls from a peer supporter. The intervention featured culturally tailored nutrition education, behavioral skills training, and social support focused on changes to diet and physical activity. The control group consisted of two 3-hour group sessions of diabetes self-management education taught by a community health worker. Outcome measures were assessed at baseline and 6 months. The primary outcome was achievement of a 5% weight reduction at 6 months. A secondary outcome was achievement of a 0.5 percentage-point reduction in hemoglobin A1c (HbA1c).

**Results:**

Groups did not differ in achievement of the weight-loss goal. Intervention participants lost a mean of 2.8 kg (*P* = .01); control participants did not lose a significant amount of weight. A greater proportion of intervention (50.0%) than control (21.4%) participants reduced HbA1c by 0.5 percentage points or more at 6 months (*P* = .03).

**Conclusion:**

The intervention was more effective than usual care (short-term diabetes education) at improving glycemic control, but not weight, in low-income African Americans with comorbid diabetes and hypertension. A community-based 6-month group class with culturally tailored education, behavioral skills training, and peer support can lead to a clinically significant reduction in HbA1c.

## Introduction

Diabetes and hypertension co-occur frequently, and both increase risk of microvascular and macrovascular complications and diabetes-related death in patients with diabetes (1). Higher prevalence of comorbid diabetes and hypertension among African Americans puts them at higher risk for diabetes complications than whites or Hispanics (2). Self-management of diabetes and hypertension requires increased physical activity and distinct dietary restrictions. Among self-management behaviors, patients find it most difficult to adhere to diet and physical activity recommendations (3). The behavioral changes necessary to control both blood glucose and blood pressure (4) are likely to be overwhelming to patients because managing both conditions is more challenging than managing either alone. Intensive, group-based interventions focused on diet and physical activity can improve weight and blood glucose control in African Americans with type 2 diabetes (5–9), but to our knowledge no study has tested an intervention designed for people with comorbid diabetes and hypertension.

This study was a pilot randomized controlled trial of an intervention to improve diet and physical activity behavior in low-income African Americans with comorbid diabetes and hypertension to reduce levels of hemoglobin A1c (HbA1c) and ultimately, long-term health risks. Our hypothesis was that an intensive group-based intervention including culturally tailored nutrition education, behavioral skills training, and social support would be more effective than usual care at motivating African Americans with comorbid diabetes and hypertension to lose weight. A secondary hypothesis was that the intervention would also result in improved glycemic control.

## Methods

### Recruitment and eligibility

For inclusion in the study, participants had to be African American, aged 18 or older, prescribed medication for type 2 diabetes and hypertension, have a body mass index (BMI [kg/m^2^]) from 25 to 45, and have no medical contraindications to participation. Patients were recruited through flyers distributed in a federally qualified health center in Chicago, Illinois. Potential participants were screened for eligibility by telephone before enrollment. Informed consent was obtained before enrollment. All procedures were approved by the Rush University Medical Center Institutional Review Board. Recruitment and follow-up took place from February 2009 through July 2010.

### Study design and intervention

The study was a randomized controlled trial comparing 2 treatments: 1) the Lifestyle Improvement Through Food and Exercise (LIFE) intervention, which was an intensive, group-based diabetes self-management class; and 2) a control treatment consisting of 2 group classes on diabetes self-management.

A class size of 15 participants was thought to be optimal for both treatments. Thus, eligible patients were not asked to provide consent until at least 30 patients were identified as willing and able to attend the classes. Two waves of 30 patients were necessary to meet the enrollment target of 60 participants, 30 per treatment group. Within each wave, participants were randomly assigned to either the intervention or control group in a 1:1 ratio so that approximately 15 patients would be assigned to each. We used a blocked randomization scheme supervised by the study statistician, alternating block sizes of 4 and 6 to ensure equal allocation to the 2 groups. Participants in each wave completed their treatments within 6 months. The 2 waves were completed within 12 months.

The LIFE intervention is grounded in 3 theoretical components that are consistent across cognitive behavioral models of behavior change (10). First, health behavior is mediated by cognitions; second, knowledge is necessary but not sufficient to produce behavior changes; and third, skills, motivation, and the social environment influence behavior change (10).

Because health behavior is mediated by cognitions, a change in dietary behavior requires a change in cognitions about food. To identify and achieve the required changes in food cognition we used the information processing model of food choice to design culturally tailored nutrition education for the LIFE intervention. This model assumes that food choice behavior can be influenced by altering the content and processing of the mental representations of food which underlie food choice decisions (11–13). The LIFE intervention aimed to teach participants how to construct meals that would most effectively control blood glucose and blood pressure; dietary guidelines were consistent with a low-sodium, moderate-carbohydrate DASH (Dietary Approaches to Stop Hypertension) diet (14,15).

The learning of new information is facilitated if it is taught in a way that is consistent with preexisting mental representations (16). The nutrition education component of the LIFE intervention was designed to integrate nutrient information into preexisting cognitive models of food to support more healthful food choices. An understanding of preexisting cognitive models of food was derived from cognitive-anthropological studies of food concepts among low-income, urban, African American women — a population similar to our target population (17,18). These studies indicated complex mental representations of food that included rich detail about how foods are eaten but rudimentary and fragmented information about nutrients (17,18). The culturally tailored nutrition education provided easy-to-understand information about nutrition and opportunities to practice applying the information via interactive activities.

Self-monitoring, goal-setting, and problem-solving skills were taught in 18 two-hour LIFE group classes (Box) and through weekly peer supporter telephone calls. The classes and telephone calls also provided emotional and social support and motivation for behavior change. Classes included a support session in which participants discussed barriers and hardships associated with chronic disease self-management and behavior change. This component was designed to create social support among participants and also develop problem-solving skills and increase self-efficacy.

**Box. Class format for Lifestyle Improvement through Food and Exercise (LIFE) intervention**

**Table:** 

Activity	Description
**1. Data collection and individual goal setting (15 min)**	**Goal setting and self-monitoring. **Weigh participants; review food logs and create individual diet goals; record pedometer steps and create individualized activity goals.
**2. Prayer (2 min) **	**Motivation. **Participant or peer supporter leads prayer.
**3. Culturally tailored educational content (45 min)**	**Nutrition education and behavioral modification. **Nutrition and diabetes education, glucose self-monitoring skills, behavioral modification techniques, interactive activities to reinforce educational content.
**4. Physical activity (10 min)**	**Social support and role modeling. **Peer supporter leads participants in moderate aerobic activity along with music.
**5. Healthy snack (15 min)**	**Nutrition education. **Healthful snack is provided and new eating behaviors are demonstrated (eg, healthy portion sizes, new healthy foods).
**6. Listening (25 min)**	**Emotional and social support, role modeling. **Participants share their struggles and victories in making behavior changes.
**7. Goal setting (10 min)**	**Goal setting and self-monitoring. **Participants set goals for activity, diet, and blood glucose monitoring for each week and discuss them with the group.

The classes were facilitated by a registered dietitian, who was assisted by 2 African American peer supporters who were selected from the community being served and had been diagnosed with either diabetes or hypertension or both. Classes were held in a local city park building near the recruitment clinic. Participants attended classes weekly for the first 3 months and every other week for the second 3 months.

For nutrition education, each participant was given a nutrition education manual, designed for the study population and including worksheets for home practice. Nutrition information was taught through interactive activities and hands-on training in such skill areas as reading food labels, portioning healthy snack foods, categorizing foods, planning meals, and counting carbohydrates.

Self-monitoring tools included daily food logs, pedometers with weekly individualized step goals, and instructions for home self-monitoring of blood glucose. Food logs were collected and steps were recorded weekly. Weekly goal setting focused on small behavioral changes in diet, physical activity, and glucose monitoring. The purpose of weekly peer supporter telephone calls was to follow up on previously set goals and address potential barriers to goal achievement.

Peer supporters trained weekly for 8 weeks (2 hours per week) with a psychologist, dietitian, and health educator. Training sessions mirrored LIFE classes to familiarize peer supporters with the nutrition education materials and prepare them to assist participants in goal setting. During training, peer supporters set their own goals and learned problem-solving skills to address barriers to goal achievement. Training and support was continued at weekly team meetings led by the study psychologist.

The control treatment consisted of two 3-hour self-management training classes taught by an African American community health worker. One class focused on diabetes self-management and the other on nutrition. The number of hours of contact time provided by the control treatment was slightly greater than the 2 hours of follow-up self-management training that is reimbursed by Centers for Medicare and Medicaid Services after initial diagnosis (19) but adequately approximates standard of care in diabetes self-management training.

### Outcome measures

Data were collected by study staff in a university clinic. Medical history, clinical variables, height and weight, medications, dietary intake, physical activity, health literacy, nutrition knowledge, and quality of life were assessed at baseline. All but medical history, health literacy, and height were reassessed at 6 months post-randomization.

#### Blood samples, blood pressure, height and weight

Trained and certified study staff collected blood samples, measured blood pressure, and assessed height and weight by using standard protocols. A whole blood sample, analyzed by Quest Diagnostics (Wood Dale, Illinois), was used to measure HbA1c. Blood pressure was measured in the resting state as the average of 3 readings taken 2 minutes apart by using an Omron digital blood pressure monitor (Omron Healthcare, Inc, Lake Forest, Illinois). Weight was measured by using a balance-beam scale; participants wore light-weight clothes and no shoes. Height was measured using a secured stadiometer. BMI was calculated by dividing weight (in kilograms [kg]) by height (in meters squared [m^2^]).

#### Self-reported variables

Self-reported sociodemographic data (sex, age, income, education) were collected through interviewer-administered questionnaires during the clinic visit. Household and leisure-time physical activity was measured as caloric expenditure per week by using the CHAMPS (Community Healthy Activities Model Program for Seniors) physical activity questionnaire modified for use among African Americans (20). Diet was assessed by using the Block Food Frequency Questionnaire (FFQ) (21), which estimated usual intake of 110 food items during the previous 3 months.

Diabetes self-management behavior was measured by using the Summary of Diabetes Self-Care Activities measure, a self-report questionnaire in which participants report on how many of the previous 7 days they performed various activities (22). Nutrition knowledge was assessed by using questions adapted from the Nutrition Knowledge Questionnaire (23); Cronbach α for the adapted scale was 0.84 at baseline and 0.80 at follow-up, and scores were calculated as proportion correct. Participants brought all medications to each visit, and medication and dosage were recorded. Adherence to diabetes and hypertension medication was measured by using the 4-item Morisky Medication-Taking Adherence Scale (24). A low rate of adherence was defined as answering yes to any of the 4 items (eg, Do you ever forget to take your medicine?). Outcome assessments were performed by staff blinded to treatment group.

### Data analyses

The primary outcome measure was the proportion of participants in each group who achieved a 5% weight loss at 6 months post-randomization. The main secondary outcome measure was the proportion in each group to achieve a 0.5 percentage-point reduction in HbA1c. Both clinical targets are associated with long-term reductions in risk of diabetes-related complications (25,26). Proportions were compared between groups by using χ^2^ tests or Fisher exact tests. Baseline characteristics were compared between groups by using *t* tests for continuous variables and χ^2^ tests for categorical variables; *t* tests were used for continuous variables to assess average change within and between groups. Wilcoxon rank-sum tests were used when *t* tests were not warranted.

The study sample size was chosen to achieve 80% power under the following assumptions: 1) 50% of participants in the LIFE group and 15% in the control group would reach goal, 2) an overall statistical significance level of .05 would be used, and 3) a 10% attrition rate would be realized. 

A sensitivity analysis assessed the effect of missing data on HbA1c and height and weight at 6 months post-randomization. The analysis consisted of 3 scenarios: 1) replacing missing values with the baseline value (ie, last observation carried forward [LOCF], indicating no treatment benefit); 2) replacing missing values in the LIFE treatment group with values indicating treatment benefit (5% reduction in weight or 0.5 percentage-point reduction in HbA1c) while replacing missing values in the control group by using LOCF; and 3) replacing missing values in the LIFE group by using LOCF while replacing missing values in the control group with values indicating treatment benefit (5% reduction in weight or 0.5 percentage-point reduction in HbA1c). Other methods for analyzing the effect of missing data, such as imputation, were not used because of limitations of the study’s small sample size. All analyses were performed by using SAS 9.2 (SAS Institute Inc, Cary, North Carolina).

## Results

We screened 183 potential participants; 61 were randomized into the study (Figure). Baseline characteristics were similar in both treatment groups (Table 1). Age ranged from 33 to 77 (mean age, 54.1 y); 70.5% had a high school education or less, and 72.2% had an annual household income of less than $20,000. Blood pressure and HbA1c were poorly controlled in approximately 30% of participants.

**Figure:**
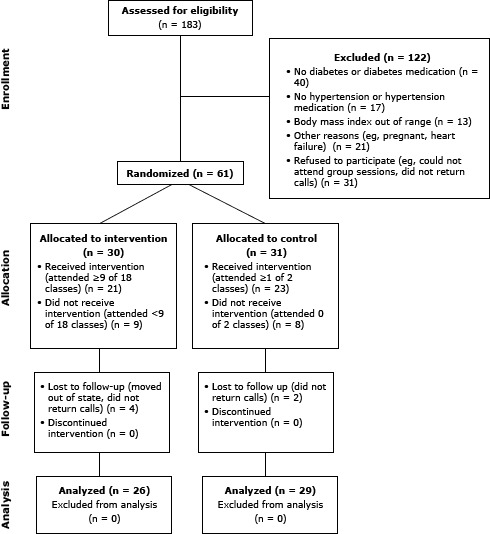
Recruitment, screening, and participation in LIFE intervention, Chicago, Illinois, 2009–2010.

**Table 1:** Baseline Characteristics of Study Participants (African American Adults With Type 2 Diabetes and Hypertension), Overall and by Group, Chicago, Illinois, 2009–2010

Characteristic	All Participants (N = 61)	LIFE Intervention (n = 30)	Control Group (n = 31)
**Age, mean (SD), y**	54.1 (10.0)	53.4 (11.4)	54.8 (8.5)
**Female, n (%)**	41 (67.2)	18 (60.0)	23 (74.2)
**Education, n (%)**			
Less than high school	16 (26.2)	7 (23.3)	9 (29.0)
High school graduate	27 (44.3)	16 (53.4)	11 (35.5)
Some college	14 (23.0)	4 (13.3)	10 (32.3)
College graduate	4 (6.5)	3 (10.0)	1 (3.2)
**Income, n (%), $**			
<10,000	30 (49.2)	13 (43.4)	17 (54.8)
10,000–19,999	14 (23.0)	9 (30.0)	5 (16.1)
20,000–35,000	10 (16.4)	4 (13.3)	6 (19.4)
>35,000	6 (9.8)	3 (10.0)	3 (9.7)
Don’t know	1 (1.6)	1 (3.3)	0
**Physical measures**			
Weight, mean (SD), lb	217.6 (47.4)	215.9 (54.0)	219.4 (40.8)
Body mass index, mean (SD), kg/m^2^	35.6 (6.3)	35.3 (6.5)	35.9 (6.3)
Systolic blood pressure, mean (SD), mm Hg	135.8 (21.4)	136.7 (23.0)	134.9 (20.2)
Diastolic blood pressure, mean (SD), mm Hg	79.6 (12.7)	82.0 (13.7)	77.3 (11.4)
Uncontrolled blood pressure, n (%)	18 (29.5)	8 (26.7)	10 (32.3)
Hemoglobin A1c, mean (SD), %	7.7 (1.6)	7.9 (1.6)	7.4 (1.6)
Hemoglobin A1c >8.0%, n (%)	16 (26.2)	9 (30)	7 (22.5)
Low rates of adherence to medications^a^, n (%)	43 (72.9)	21 (70.0)	22 (75.9)
**Health measures**			
Age at diabetes diagnosis, mean (SD), y	45.4 (10.3)	45.0 (11.4)	45.8 (9.3)
Takes oral medications for diabetes, n (%)	50 (82.0)	24 (80.0)	26 (83.9)
Uses insulin for diabetes, n (%)	26 (42.6)	13 (43.3)	13 (41.9)
**Dietary measures, mean (SD)^b^**			
Dietary intake, kcal/d	2,287.0 (1,741.5)	2,386.5 (2,035.7)	2,190.9 (1,430.1)
Dietary sodium intake, mg/d	4,099.2 (3,010.4)	4,147.0 (3,371.7)	4,053.0 (2,672.9)
Fat intake, % of total intake	39.8 (6.1)	40.0 (6.2)	39.7 (6.2)
Protein intake, % of total intake	17.5 (3.7)	17.4 (3.2)	17.7 (4.2)
Carbohydrate intake, mean (SD), % of total intake	42.7 (6.8)	42.6 (6.6)	42.6 (7.1)
**Physical activity, mean (SD), kcal/wk^c^**			
Total activity	4,502.1 (3,592.6)	3,943.7 (2,679.8)	5,127.5 (4,371.9)
Moderate/vigorous activity	1,714.6 (2,210.6)	1,386.0 (1,665.6)	2,082.7 (2,683.3)
**Medical history, n (%)**			
Smoker	18 (30.0)	12 (40.0)	6 (20.0)
Arthritis	33 (54.1)	13 (43.3)	20 (64.5)
Depression	20 (32.8)	12 (40.0)	8 (25.8)
Treatment for depression	16 (26.2)	10 (33.3)	6 (19.4)
**Nutrition knowledge, mean % of correct answers^d^**	60 (15)	57 (19)	63 (11)
**Performed diabetes self-care activities, no. of days in past week** e			
General diet	3.3 (2.4)	3.1 (2.8)	3.5 (2.0)
Specific diet	3.6 (1.9)	3.8 (2.0)	3.5 (1.9)
Exercise	2.5 (2.0)	2.5 (2.0)	2.6 (2.0)
Glucose testing	3.5 (1.8)	3.6 (2.1)	3.4 (1.4)

Abbreviations: LIFE, Lifestyle Improvement through Food and Exercise; SD, standard deviation.

a Assessed by using the 4-item Morisky Medication-Taking Adherence Scale (24). A low rate of adherence was defined as answering yes to any of the 4 items (eg, Do you ever forget to take your medicine?).

b Assessed by using the Block Food Frequency Questionnaire (21), which estimated usual intake of 110 food items during the previous 3 months.

c Assessed as caloric expenditure per week by using a CHAMPS (Community Healthy Activities Model Program for Seniors) physical activity questionnaire modified for use among African Americans (20).

d Assessed by using questions adapted from the Nutrition Knowledge Questionnaire (23).

e Assessed by using the Summary of Diabetes Self-Care Activities measure (22). Reference values derived from 7 studies of white adult type 2 diabetes patients are as follows: general diet, 4.1 days; specific diet, 4.7 days; exercise, 2.4 days; glucose testing, 4.8 days (27).

Fifty-five participants (90%) completed the 6-month follow-up. LIFE participants attended an average of 11.2 (standard deviation [SD], 6.3, median, 14) of 18 classes. Peer supporters successfully completed 53% of attempted telephone calls. Control participants attended an average of 1.3 of 2 classes.

At 6 months post-randomization, we found no significant difference between treatment groups in the proportion of participants who lost 5% or more of their body weight: of 26 LIFE participants, 7 (26.9%) lost 5% or more of their body weight; of 29 control participants, 7 (24.1%) did so (*P* = .81 for difference between proportions). However, a greater proportion of LIFE participants than control participants achieved a reduction in HbA1c of 0.5 percentage points or more: 13 (50.0%) LIFE participants and 6 (21.4%) control participants (*P* = .03 for difference in proportions). Four LIFE participants and 1 control participant had missing 6-month values for both BMI and HbA1c, and 1 control participant was missing only the 6-month HbA1c value. The use of the LOCF strategy and that of replacing missing values in the LIFE group with values indicating treatment benefit (while using the LOCF strategy in the control group) yielded *P* values similar to those above. Replacing missing values in the control treatment group with values indicating treatment benefit (while using the LOCF strategy in the LIFE treatment group) yielded a similar *P* value for the weight-loss comparison and a *P* value of .25 for the HbA1c comparison.

The difference in mean weight change between treatment groups at 6 months (−1.8 kg) was not significant (Table 2). LIFE participants showed significant mean weight loss at 6 months (−2.8 kg, *P* = .01), but control participants did not (−1.1 kg, *P* = .21). The LIFE group achieved, on average, the targeted reduction of 0.5 percentage points in HbA1c at 6 months (*P* = .05). The reduction of HbA1c in the LIFE group was not significantly different (*P* = .10) from the nonsignificant increase in the control group.

**Table 2:** Mean Change in Study Variables From Baseline to 6 Months, By Group, for African American Adults With Type 2 Diabetes and Hypertension, Chicago, Illinois, 2009–2010

Measure	LIFE Intervention (n = 26)	Control Group (n = 29)	Difference (95% CI)	*P* Value
**Clinical measures**				
Weight, kg	−2.8	−1.1	−1.8 (−4.3 to 0.8)	.17
Hemoglobin A1c, %	−0.5	0.1	−0.6 (−1.2 to 0.1)	.10
Systolic blood pressure, mm Hg	−3.6	0.1	−3.7 (−15.8 to 8.4)	.54
Diastolic blood pressure, mm Hg	−2.7	1.0	−3.7 (−10.0 to 2.7)	.26
**Performed diabetes self-care activities, no. of days in past week^a^**				
General diet	2.3	0.5	1.9 (0.6 to 3.1)	<.001
Specific diet	1.5	0.3	1.2 (0.2 to 2.2)	.02
Exercise	1.5	0.6	0.8 (−0.4 to 2.0)	.17
Glucose testing	0.4	−0.3	0.8 (−0.3 to 1.9)	.15
**Dietary measures^b^**				
Dietary intake, mean (SD), kcal/d	−109.8	−247.9	138.1 (−676.2 to 952.3)	.74
Dietary sodium, mg/d	−171.3	−475.9	304.6 (−1,005.1 to 1,614.0)	.64
Protein intake, % of total intake, percentage point	1.3	−1.1	2.4 (0.7 to 4.2)	.01
Carbohydrate intake, % of total intake, percentage point	0.7	1.7	−0.9 (−4.6 to 2.9)	.64
Fat intake, % total intake, percentage point	−2.0	−0.4	−1.6 (−4.8 to 1.8)	.34
Vegetables, servings/d	1.3	−0.4	1.7 (−0.01 to 3.3)	.05
Fiber, g/d	2.1	−2.3	4.4 (−1.9 to 10.8)	.16
Cholesterol, mg/d	−6.5	−53.0	46.5 (−77.1 to 170.1)	.45
Potassium, mg/d	92.9	−375.6	468.5 (−467.1 to 1,404.2)	.32
**Physical activity, kcal/wk^c^**	1,913.6	−603.4	2,517.1 (590.3 to 4,443.8)	.01
**Nutrition knowledge^d^, mean % correct answers, percentage point**	18.0	7.6	10.4 (2.3 to 18.4)	.01

Abbreviations: LIFE, Lifestyle Improvement Through Food and Exercise; CI, confidence interval; SD, standard deviation.

a Assessed by using the Summary of Diabetes Self-Care Activities measure (22). Reference values derived from 7 studies of white adult type 2 diabetes patients are as follows: general diet, 4.1 days; specific diet, 4.7 days; exercise, 2.4 days; glucose testing, 4.8 days (27).

b Assessed by using the Block Food Frequency Questionnaire (21), which estimated usual intake of 110 food items during the previous 3 months.

c Assessed as caloric expenditure per week by using a CHAMPS (Community Healthy Activities Model Program for Seniors) physical activity questionnaire modified for use among African Americans (20).

d Assessed by using questions adapted from the Nutrition Knowledge Questionnaire (23).

Among control participants from baseline to 6-month follow-up, 5 (17%) reduced and 7 (24%) increased either the dosage or the number of diabetes medications; among LIFE participants, 7 (27%) reduced and 3 (12%) increased their medications. We found no changes in adherence to medications for either group.

Although we found reductions at 6 months in both systolic and diastolic blood pressure in the LIFE group, they were not significant compared with baseline or compared with the nonsignificant increases in the control group (Table 2).

In the performance of diabetes self-management behaviors, the LIFE group reported significantly greater improvement than the control group in the number of days they followed a general diet (difference between 2 groups = 1.9 days; *P* < .001) and in the number of days they followed a specific diet (difference between 2 groups = 1.2 days; *P* = .02) (Table 2). The LIFE group reported significant improvement in exercise behavior from baseline to follow-up (*P* = .004), but this increase was not significantly different from the nonsignificant decrease in the control group (Table 2).

The LIFE group reported a significantly greater increase in percentage of calories from protein than the control group (a difference of 2.4 percentage points) (Table 2). Changes in the other dietary measures were not significant within or between groups.

The LIFE group showed a significantly larger increase in physical activity than the control group (a difference of 2,517.1 kcal/week) (Table 2). The LIFE group, but not the control group, showed a significant increase in physical activity at 6 months relative to baseline (*P* = .006). Both groups improved their nutrition knowledge from baseline to follow-up, but the LIFE intervention group showed a significantly greater increase (10.4 percentage points more than the control group) (Table 2).

## Discussion

This pilot study evaluated the effectiveness of a newly developed behavioral intervention to reduce weight in disadvantaged African Americans with comorbid diabetes and hypertension. LIFE and control participants were equally likely to achieve the target 5% weight loss. LIFE participants achieved a significant weight loss relative to baseline (−2.8 kg), but the study was not powered to detect this difference. The amount of weight loss in the LIFE group was consistent with weight loss achieved by healthy African American participants in other group-based behavioral weight loss trials (range from 0.05 to −4.7 kg) (27).

LIFE participants were 2.2 times as likely as control participants to achieve a clinically significant reduction in HbA1c. Recent findings suggest that weight reduction may not result in long-term reduction in cardiovascular disease among patients with type 2 diabetes (28), although much evidence shows that reduction in HbA1c results in decreased microvascular complications (25). Thus, despite the lack of significant weight loss, the LIFE intervention could potentially reduce long-term risk from diabetes complications.

Many behavior changes were associated with the intervention. The LIFE group at 6 months showed greater improvement than the control group in the number of days they ate a healthy diet, a greater percentage of daily calories from protein, a greater increase in caloric expenditure from physical activity, and a greater increase in knowledge of diabetes nutrition.

Similar to the other successful diabetes self-management intervention trials with African Americans (6,7,9), our study was limited by a small sample size and a short follow-up period. The small sample size of our study compromised our ability to detect meaningful changes in secondary outcomes. A strength of this study is that we ruled out the possibility of confounding due to differences in medication use.

The control group in our study received an intervention that is arguably more intensive than usual care because it provided more hours of class time taught by a community health worker to increase cultural tailoring. More hours of diabetes education as well as cultural tailoring are associated with greater improvements in HbA1c (29). Thus, the potential strength of the LIFE intervention relative to usual care may have been underestimated.

Another limitation of this study is use of the Block FFQ to measure changes in dietary intake. The FFQ is an appropriate tool to measure change in interventions (30), but some of our participants had difficulty answering some of the questions, and approximately one-third of our sample reported daily caloric intakes that were less than 500 kcal or greater than 5,000 kcal. These factors raise questions about the validity of this FFQ as a measure of dietary change in this population.

Lifestyle changes were achieved in a high-risk population of urban African Americans. This pilot study showed that, compared with short-term group-based diabetes self-management education (usual care), a community-based group class featuring appropriately tailored education and strong behavioral support, supplemented with individual peer support, can lead to a clinically significant reduction in HbA1c. If sustained, these behavioral and physiological changes can be expected to result in long-term reduced risk of diabetes complications and mortality among patients with varying levels of glycemic control. Long-term effectiveness of this intervention is being examined in a larger sample of low-income African American diabetes patients.
